# MicroRNAs and Cancer Racial Disparities

**DOI:** 10.1002/wrna.70028

**Published:** 2025-09-25

**Authors:** Dan Zhao, Yifei Wang

**Affiliations:** ^1^ Section of Epidemiology and Population Sciences, Department of Medicine Baylor College of Medicine Houston Texas USA; ^2^ Dan L Duncan Comprehensive Cancer Center, Baylor College of Medicine Houston Texas USA

**Keywords:** cancer racial disparities, microRNAs, miR genetic variations

## Abstract

Cancer remains one of the leading causes of death worldwide. Despite various efforts to reduce cancer mortality, such as decreasing tobacco use, improving early detection and prevention methods, and enhancing cancer care and treatments, certain racial and ethnic groups continue to experience higher cancer incidence and mortality rates, along with shorter survival compared to other groups. Several factors, including socioeconomic status, environmental influences, diet, and behavior, contribute to these racial disparities. More importantly, scientists have identified a genetic basis for these observations, with a growing body of research highlighting microRNAs as significant players in cancer racial disparities. This review focuses on various types of microRNAs (such as epigenetically regulated, copy number altered, circulating, and exosomal) and microRNA single‐nucleotide variations in the context of cancer‐related racial disparities. Additionally, we have summarized the existing resources, including racial‐specific model cell lines and cancer cohorts that include patients from diverse racial and ethnic backgrounds. Moreover, we provide here several key things to consider for future investigations. While many challenges remain, we aim to offer a balanced overview of this field to help scientists with varying expertise address these issues.

This article is categorized under:

RNA in Disease and Development > RNA in Disease

## Graphical abstract

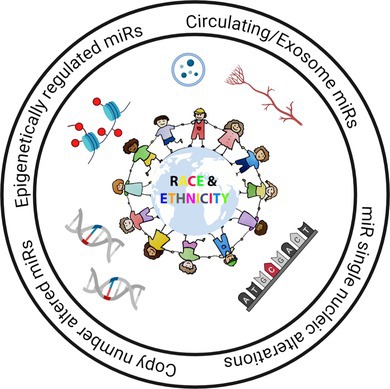

## 1 Introduction

### 1.1 Overview of Cancer Racial Disparities

Cancer is the second leading cause of death worldwide, with an estimated 18.1 million new cases and 9.6 million cancer‐related deaths in 2018. The burden on individuals, families, and society is immense, with the annual economic cost of cancer reaching US$1.16 trillion (Wild et al. 2020). Despite global cooperation and the collective efforts of many scientists, statistics indicate that certain racial groups are disproportionately affected by cancer in terms of incidence, mortality, and aggressiveness. Numerous studies comparing African Americans (AA) and Caucasian Americans (CA) provide solid evidence of this disparity. Overall, AAs have higher death rates from all cancers combined compared to other groups. For instance, cancer statistics from 2019 show that AAs have higher incidence rates for kidney, liver, prostate, stomach, uterine cervix, colorectal, pancreatic cancers, Kaposi sarcoma, and myeloma. Additionally, AAs with breast, liver, colorectal, prostate, uterine cervix, pancreatic, endometrial cancer, and myeloma exhibit higher mortality rates and shorter survival times.

It is widely accepted that cancer racial disparity is a multifactorial issue, influenced by a combination of factors such as socioeconomic status, culture, environmental influences, behavior, and access to early detection, prevention screenings, and treatment. However, studies have also clearly shown a genetic component for some types of cancer. For example, certain cancer‐causing genetic mutations are more prevalent in AAs compared to CAs, and some genes are specifically overexpressed in AAs (Daly and Olopade 2015; Ozdemir and Dotto 2017; Wallace et al. 2011).

### 1.2 There Is a Lack of Available Resources for Cancer Racial Disparity Research

While epidemiological evidence is clear for cancer racial disparities, the knowledge of its causes is still emerging. Researchers face the challenge of limited resources to study racial disparities in human cancers. As summarized in Table 1, the currently available cell line models (Dutil et al. 2019; Kessler et al. 2019) for different cancer types show that most of the currently available cancer cell line models are derived from Caucasian/White patients, and those from AA, Asian, Hispanic, or other races/populations are only limitedly available for a few cancer types. Additionally, we summarized the patient numbers in cBioPortal (Cerami et al. 2012; Gao et al. 2013) for TCGA pan‐cancer cohorts, the world's largest and most prominent cancer‐specific multi‐omics data resources, with known race/ethnicity information. However, only five cancer types have more than 50 non‐Hispanic Black (NHB) patients available, and four cancer types have more than 50 Asian patients; unfortunately, none of them include more than 50 Hispanic patients (Table 2). This identifies a significant under‐representation of racial and ethnic minority populations in cancer research and presents a great challenge to understand cancer population genetics and precision medicine.

**TABLE 1:** Cancer cell lines from different racial/ethnic backgrounds.

Cancer tissue origin	African/Black	Caucasian/White	Undefined	Lines of other ethnicities
Adrenal gland	NA	1	NA	NA
Autonomic ganglia	NA	11	26	2 Japanese
Biliary tract	NA	1	9	2 Japanese
Bone	1	20	34	1 Turkish and 5 Japanese
Breast	9	39	11	1 French, 1 Indian, 2 Japanese, and 1 Hispanic
Cervix	1	7	3	1 Mongoloid and 3 Japanese
CNS	1	18	57	3 Japanese and 6 Mongoloid
Endometrium	NA	7	4	16 Japanese and 2 Mongoloid
Hematopoietic and lymphoid	11	61	141	1 American and 36 Japanese
Kidney	NA	12	29	9 Japanese, 1 Chinese, and 2 Mongoloid
Large intestine	NA	30	25	1 American Indian, 3 Asian, 1 Japanese, and 9 Mongoloid
Liver	1	3	6	9 Japanese, 4 Korean, and 3 Mongoloid
Lung	13	113	83	21 Japanese
Esophagus	1	6	17	1 Asian and 13 Japanese
Ovary	1	11	33	16 Japanese and 3 Mongoloid
Pancreas	NA	21	14	11 Japanese and 3 Mongoloid
Placenta	NA	1	NA	NA
Pleura	NA	6	20	1 Japanese
Prostate	1	6	2	1 Japanese
Salivary gland	NA	1	NA	1 Mongoloid
Skin	NA	28	51	1 Asian, 1 European, and 4 Japanese
Small intestine	NA	1	NA	NA
Soft tissue	1	13	9	2 Japanese
Stomach	NA	2	12	1 Asian, 1 Hispanic, 18 Japanese, 3 Korean, and 6 Mongoloid
Testis	NA	1	NA	2 Japanese
Thyroid	NA	3	10	1 Chinese and 4 Japanese
Upper aerodigestive tract	NA	14	28	8 Japanese and 7 Mongoloid
Urinary tract	2	11	12	1 Chinese and 1 Japanese
Vulva	NA	2	1	NA

*Note:* Ethnicity information for cell lines from CCLE (Cancer Cell Line Encyclopedia) was summarized and subgrouped by their tissue origin.

Abbreviation: NA, not available.

**TABLE 2:** Number of cancer patients from different racial/ethnic backgrounds in TCGA.

Cancer type	Number of NHW	Number of NHB	Number of Asia	Number of Hispanic or Latino
Breast cancer	651	166	60	38
Non‐small cell lung cancer	588	80	17	15
Glioma	424	16	7	32
Melanoma	411	1	10	10
Head and neck cancer	405	42	11	26
Bladder cancer	308	17	43	9
Renal clear cell carcinoma	291	49	8	25
Endometrial cancer	277	74	20	15
Colorectal cancer	276	56	12	5
Thyroid cancer	270	26	52	38
Esophagogastric cancer	267	10	135	11
Ovarian epithelial tumor	229	10	17	7
Renal non‐clear cell carcinoma	201	58	8	16
Glioblastoma	194	27	4	6
Sarcoma	193	18	6	5
Hepatobiliary cancer	158	17	160	17
Prostate cancer	144	6	2	0
Cervical cancer	127	17	20	23
Pancreatic cancer	126	7	11	5
Pheochromocytoma	94	13	5	5
Thymic epithelial tumor	81	6	13	9
Pleural mesothelioma	71	1	1	0
Non‐seminomatous germ cell tumor	54	1	1	7
Seminoma	48	2	3	5
Adrenocortical carcinoma	38	0	2	8
Cholangiocarcinoma	28	2	3	2
Miscellaneous neuroepithelial tumor	19	6	1	0
Mature B‐cell neoplasms	17	1	18	12

*Note:* Number of patients for ethnicity specified from each cancer type from the TCGA pan‐cancer cohorts. Data from cBioPortal.

Abbreviations: NHB, non‐Hispanic Black; NHW, non‐Hispanic White.

### 1.3 microRNAs and Cancer Racial Disparities

MicroRNAs are small noncoding RNAs, approximately 20 nucleotides long, that are derived from primary transcripts and processed canonically through a two‐step cleavage by the enzyme complexes Drosha and Dicer. The first step, known as cropping, produces stem‐loop‐structured precursor microRNAs. These precursors, or pre‐miRNAs, are then further cleaved by the enzyme Dicer in a process called dicing, resulting in mature microRNAs. Atypically, microRNA biogenesis happened independently of Dicer or Drosha (O'Brien et al. 2018; Shang et al. 2023). These mature microRNAs are subsequently incorporated into the RNA‐induced silencing complex (RISC).

Since the discovery of the first microRNA in 1993, the number of identified microRNAs has dramatically increased over the past two decades. According to the most recent miRBase release (V22.1), there are currently 1917 precursor and 2654 mature microRNAs in humans (Kozomara and Griffiths‐Jones 2014). Most microRNAs are believed to be negative regulators that bind to the 3′‐untranslated region (3′‐UTR) of target genes, leading to either degradation of the mRNA or inhibition of translation. Despite their small size, microRNAs are significant players in cancer research and are implicated in nearly all types of human cancers (Sempere et al. 2021). They contribute to tumor initiation, progression, and therapy resistance, and there is a growing body of research linking microRNAs to cancer racial disparities. Given the recent Nobel Prize awarded to Drs. Victor Ambros and Gary Ruvkun for their groundbreaking discovery of microRNAs, we find it timely to provide an in‐depth summary of the current knowledge in this field. In this review, we summarize the role of microRNAs in cancer racial disparities by focusing on (i) copy number altered microRNAs, (ii) epigenetically regulated microRNAs, (iii) circulating and exosomal microRNAs, and (iv) other microRNAs (Table 3) as well as microRNA‐related single‐nucleotide variations (SNVs) (Table 4). To maintain fidelity to the original studies cited and honor the authors' chosen classifications and maintain consistency with their reported findings, we have preserved terminology such as AA, NHB, CA, European American (EA), and non‐Hispanic White (NHW), acknowledging that these classifications often overlap in definitions. Above all, this review affirms the critical connections between microRNAs and cancer‐related racial disparities, reinforcing their role as molecular contributors to differential outcomes across populations. Furthermore, throughout the manuscript, we have adhered to the nomenclature guidelines established by the HUGO Gene Nomenclature Committee (Seal et al. 2020) to ensure consistency, evolutionary accuracy, and clarity in microRNA annotation.

**TABLE 3:** MicroRNAs and cancer racial disparities.

Category	MIRNA ID	Cancer type	Race/ethnicity	Source	Country study done	PMID or DOI
Copy number altered microRNAs	*MIR4288*	PC	AA and CA	74 TN pairs of PC including 39 AA and 21 CA	USA	30874288 (Bhagirath et al. 2019)
	*MIR342*	TNBC	AA and CA	259 breast tumors	USA	21264507 (Loo et al. 2011)
	*MIR151*	PC	AA	Total of 37 patients	USA	21456068 (Barnabas et al. 2011)
	*Multiple*	TNBC	AA and NHW	27 AA TNBC and 30 NHW TNBC	USA	27813494 (Sugita et al. 2016)
	*MIR34B*	PC	AA and CA	81 AA and 62 CA, methylation data from TCGA	USA	28039468 (Shiina et al. 2017)
Epigenetically regulated microRNAs	*MIR24*	PC	AA and CA	81 AA and 51 CA patients	USA	28157714 (Hashimoto et al. 2017)
	*9 MIRs*	CRC	AA and CA	6 AA TN pair and 7 CA TN pair	USA	27111221 (Wang et al. 2016)
	*MIR152*	PC	AA and CA	5 CA and 6 AA lines; 20 AA and 19 CA patients	USA	25004396 (Theodore et al. 2014)
	*MIR34B*	PC	AA and CA	81 AA and 62 CA, methylation data from TCGA	USA	28039468 (Shiina et al. 2017)
Circulating or exosomal microRNAs	miR‐125b, miR‐155, and miR‐3613	PC	AA and CA	Both cell lines and patients' samples, N not specified	USA	https://doi.org/10.1158/1538‐7445.AM2016‐1775 (Moustafa et al. 2016)
	miR‐21	Multiple	Asian and Caucasian	Meta‐analysis of 36 studies on 15 cancer types involving 2920 cases and 1986 controls	China	25527152 (Wu et al. 2015)
	miR‐101	PC	AA and CA	Serum of 12 normal AA vs. 24 AA PC and 20 normal CA vs. 16 CA PCs	USA	24477576 (Srivastava et al. 2014)
	miR‐1304‐3p	BC	AA and CA	19 AA and 20 CA serum	USA	36517516 (Zhao et al. 2022)
	miR‐510‐5p	BC	AA and CA	9 AA and 10 CA serum	USA	37822942 (King et al. 2023)
	Multiple	OC	NHW and others	1220 non‐Hispanic Whites vs. 366 others	USA	38388186 (Alimena et al. 2024)
	Multiple	Early‐stage BC	AA and CA	10 CA and 10 AA tumor and normal plasma samples	USA	21060830 (Zhao et al. 2010)
	Multiple (miR‐155)	Early‐stage NSCLC	AA and CA	Serum and plasma samples from 220 tumor patients (177 CA + 43 AA) and 220 healthy (171 CA + 49 AA) controls	USA	21544802 (Heegaard et al. 2012)
Other microRNAs	Multiple	CRC	AA and CA	3 AA and 3 CA CRC cell lines	USA	30066857 (Paredes et al. 2018)
	*MIR17*	Multiple	Asian and non‐Asian	Meta of 12 studies involving 1096 patients	China	29858404 (Huang et al. 2018)
	*MIR181B*	CRC	AA and CA	TN pairs from 106 AA and 239 CA patients	USA	23719259 (Bovell et al. 2013)
	*MIR182*	CRC	AA and CA	30 AA and 31 CA patients	USA	24865442 (Li et al. 2014)
	MIR‐200 family	Multiple	Multiple	Meta‐analysis of 28 studies involving 2097 patients and 1579 controls	China	26618619 (Liu et al. 2016)
	*MIR200C*	Leiomyoma	Multiple	76 TN pairs	USA	22685266 (Chuang et al. 2012)
	*MIR212*	PC	AA and CA	13 AA and 17 CA cancer tissues	USA	26553749 (Y. Yang, Jia, et al. 2016)
	*MIR221* and *MIR31*	PTC	AA and CA	14 CA and 8 AA and adjacent normal	USA	26380656 (Suresh et al. 2015)
	*MIR29*	Multiple	Multiple	Meta‐analysis of 20 studies involving 1966 patients	China	28063172 (Qi et al. 2017)
	*MIR494*	Multiple	Asian and CA	Meta‐analysis of 15 studies of 1104 patients	China	29416694 (Xiang et al. 2018)
	*MIR99B*	PC	AA and CA	15 CA and 25 AA and adjacent normal	USA	24167554 (Srivastava et al. 2013)
	Multiple	EC	Black and White	Discovery cohort: 50 (9 B and 41 W), validation cohort: 47 (24 B and 23 W) patients	USA	25174797 (Maxwell et al. 2015)
	Multiple	NSCLC	AA and EA	42 AA and 55 EA patients	USA	29196495 (Mitchell et al. 2017)
	Multiple	Early‐stage BC	Lebanese and American	45 Lebanese and 197 matched American BC patients.	USA	29203780 (Nassar et al. 2017)
	Multiple	BC	British Caucasian, British Black, Nigerian, and Indian	A total of 17 patients 5 British Caucasian, 4 each for the other 3 groups	UK	29791912 (Pollard et al. 2018)
	Multiple	PC	AA and CA	TN pairs from 20 AA and 15 CA	USA	26089375 (Wang et al. 2015)
	*MIR4719* and *MIR6756*	PC	AA and CA lines	1 AA line and 2 CA lines	USA	30669553 (Paredes et al. 2018)
	Multiple	EC	AA, CA, and Asian	374 CAs, 109 AAs and 20 Asians	UK	29682207 (Guttery et al. 2018)
	Multiple	Gynecologic cancers	AA and CA	305 AAs and 1402 CAs	USA	35992327 (Asare et al. 2022)

*Note:* Key papers on microRNAs implicated in human cancer racial disparities.

Abbreviations: AA, African American; CA, Caucasian American; CRC, colorectal cancer; EA, European American; EC, endometrial cancer; NHW, non‐Hispanic White; NSCLC, non‐small cell lung cancer; OC, ovarian cancer; PC, prostate cancer; PTC, papillary thyroid carcinomas; TN, Tumor/Normal; TNBC, triple‐negative breast cancer.

**TABLE 4:** MicroRNA‐related genetic variants in cancer racial disparities.

	SNVs	Cancer type	Race/ethnicity studied	Racial‐specific effect in	Source	Country study done	PMID
MIR targets UTR SNPs	rs8176318 in BRCA1 UTR	BC	AA and EA	AA	102 AA and 92 EA patients	USA and China	21191178 (Pelletier et al. 2011) and 27073502 (Pelletier et al. 2011; F. Yang, Chen, et al. 2016)
	rs712 in KRAS UTR	Multiple	Chinese and Caucasian	Chinese	Meta‐analysis of 6 studies (5 different types of cancer) involving > 1500 patients and > 2500 healthy controls	China	25210463 (Ying et al. 2014)
	rs34149860 in STAG1 UTR	CRC	AA	AA	95 AA patients	USA	29471289 (Datta et al. 2018)
	rs1131445 at IL16 UTR	PC	AA and CA	AA	256 AA patients and 207 CA patients	USA	24061634 (Hughes et al. 2013)
MIR SNPs	MicroRNAs SNPs	Multiple	AA and non‐AA (total of 14 populations)	AA	69 individuals from 14 populations	USA	25169894 (Rawlings‐Goss et al. 2014)
	*MIR146A* rs2910164	HCC	Turkish	No association	222 cancer 222 control	Turkey	21807077 (Akkiz et al. 2011c)
	*MIR146A* rs2910164	GC	Caucasian and Asian	Asian	Meta‐analyses of 8 case–control studies involving 4308 cases and 6370 controls	Sweden	25455160 (Fu et al. 2014)
	*MIR146A* rs2910164	CC	Chinese Han and Uygur	GG/CG genotypes are significantly correlated with Uygur and larger tumors	208 control, 207 cervical intraepithelial neoplasia (CIN), and 205 cervical cancer samples	China	26464690 (Ma et al. 2015)
	*MIR146A* rs2910164	PC	European Caucasian and Asian	Asian but not Caucasian	Meta‐analysis of 5 studies	Japan	30001553 (Mi et al. 2018)
	*MIR146A* rs2910164	HCC	Multiple	Asian but not Caucasian	Meta‐analysis of 12 studies including 4171 cases and 4901 controls	China	25546664 (Peng et al. 2014)
	*MIR146A* rs2910164	HCC	Chinese only	No association	172 patients and 185 controls	China	24301908 (Shan et al. 2013)

	*MIR146A* rs2910164	Multiple	Asian and Caucasian	Caucasian	Meta‐analysis of 18 studies (12 Asian cohorts + 8 Caucasian cohorts) involving 9207 cases and 11,453 controls	China	22952151 (J. Wang, Wang, et al. 2012)
	*MIR146A* rs2910164 and *MIR196A2* rs11614913	HCC	Asian	No association with HCC	Meta‐analysis of 5 studies	China	22768213 (Z. Wang, Cao, et al. 2012)
	*MIR196A2* rs11614913	CRC	Iranian	Iranian	2150 Iranian patients and meta‐analyses	Iran	29802998 (Haerian et al. 2018)
	*MIR196A2* rs11614913	HCC	Turkish	Turkish	185 TN pair	Turkey	21692953 (Akkiz et al. 2011a)
	*MIR196A2* rs11614913	Glioma	Chinese	Chinese	670 cases and 680 controls	China	20229273 (Dou et al. 2010)
	*MIR196A2* rs11614913	BC	CA	No association	193 bc and 190 controls	Australia	21962133 (Jedlinski et al. 2011)
	*MIR196A2* rs11614913	Multiple	Multiple	Asian	Meta of 46 studies involving 20,673 cases and 25,143 controls	China	24633889 (Kang et al. 2014)
	*MIR196A2* rs11614913	PC	Caucasian and Asian	Asian	Meta‐analysis of 3 studies	Japan	30001553 (Mi et al. 2018)
	*MIR196A2* rs11614913	HCC	Multiple	No association	Meta‐analysis of 12 studies including 4171 cases and 4901 controls	China	25546664 (Peng et al. 2014)
	*MIR196A2* rs11614913	Multiple	Asian and Caucasian	Asian	Meta‐analysis of 9 studies involving 6540 cases and 7562 controls	China	21625865 (F. Wang, Ma, et al. 2012)
*MIR196A2* rs11614913	Multiple	Asian and Caucasian	Asian	Meta‐analysis of 21 studies (16 Asian cohorts + 5 Caucasian cohorts) involving 11,764 cases and 14,254 controls	China	22952151 (J. Wang, Wang, et al. 2012)

	*MIR27A* rs895819	CRC	Chinese	Chinese	508 cases and 562 controls	China	26302683 (Jiang et al. 2016)
	*MIR27A* rs895819	BC and OC	Jewish	Jewish	125 BRCA2 mutation carriers	Israel	19950226 (Kontorovich et al. 2010)
	*MIR1304* rs2155248	Multiple	AA	AA	TCGA	USA	36517516 (Zhao et al. 2022)
	*MIR423* rs6505162	BC	Chilean	Chilean	440 cases and 807 controls	Chile	27421647 (Morales et al. 2016)
	*MIR423* rs6505162	OC	Jewish	Jewish	125 BRCA2 mutation carriers	Israel	19950226 (Kontorovich et al. 2010)
	*MIR499* rs3746444	HCC	Turkish	No association	222 cancer 222 control	Turkey	22393998 (Akkiz et al. 2011b)
	*MIR499* rs3746444	Multiple	Multiple	Asian	Meta‐analyses of 31 case–control studies involving 12,799 cases and 14,507 controls	China	25433484 (Chen et al. 2014)
	*MIR499* rs3746444	PC	Caucasian and Asian	Asian	Meta‐analysis of 3 studies	Japan	30001553 (Mi et al. 2018)
	*MIR499* rs3746444	HCC	Chinese	Chinese	172 patients and 185 controls	China	24301908 (Shan et al. 2013)
	*MIR499* rs3746444	Multiple	Asian and Caucasian	Asian	Meta‐analysis of 65 case–control studies involving 23,762 cases and 28,694 controls	China	29946268 (Yang et al. 2018)
*MIR608* rs4919510	CRC	AA and CA	Increased risk of death in CA but reduced risk in AA	245 cases and 446 controls	USA	22606253 (Ryan et al. 2012)

Multiple SNPs	BC	AA and CA	rs7354931 in AGO4, rs12586258 in *MIR‐758*, and rs2018562 in *MIR‐513A2* associated with AA BC; rs2059691 in *PACT*, rs1527423 in *MIR‐106B*, rs1834306 in *MIR‐100*, rs11107973 in *MIR‐331*, rs10144193 in *MIR‐544*, rs1951032 in *MIR‐487*, rs5750504 in *MIR‐659* with CA BC	906 AA and 653 EA	USA	24062209 (Yao et al. 2013)
IsomiRs	isomiRs	TNBC	AA and CA		66 CA and 32 AA cancer patients, 94 CA and 6 AA normal	USA	29229607 (Telonis and Rigoutsos 2018)
	isomiRs	PC	AA and CA		526 samples from 472 patients	USA	29593348 (Magee et al. 2018)
	isomiRs	TNBC	AA and CA		316 samples from TCGA	USA	26400174 (Telonis et al. 2015)
	isomiRs	Lymphoblastoid cell lines	Five different populations		452 lymphoblastoid cell lines	USA	25229428 (Loher et al. 2014)

*Note:* Key papers on microRNAs related to genetic variants implicated in human cancer racial disparities.

Abbreviations: AA, African American; BC, breast cancer; CA, Caucasian American; CC, cervical cancer; CRC, colorectal cancer; EA, European American; GC, gastric cancer; HCC, hepatocellular cancer; OC, ovarian cancer; PC, prostate cancer; TCGA, The Cancer Genome Atlas; TNBC, triple‐negative breast cancer.

### 1.4 Copy Number Altered microRNAs in Cancer Racial Disparities

In the early 2000s, scientists began to explore microRNAs, a class of small noncoding RNAs that are approximately 20 nucleotides long. They discovered that these RNAs are nonrandomly scattered throughout the human genome, with more than half located at fragile sites and cancer‐associated regions (Calin et al. 2004) and are either amplified or depleted. With over 2000 microRNAs identified in the human genome to date, it is not surprising that some of them are linked to cancer in a race‐specific manner and contribute to racial disparities.

One study analyzing 74 prostate cancer (PC) tumor/normal pairs (39 AA and 21 CA) found that the *MIR4288* locus in the 8q21 region is specifically depleted in CAs but not in AAs, leading to epithelial–mesenchymal transition (EMT) by de‐repressing MMP16 and ROCK1 (Bhagirath et al. 2019). Another study involving 259 young breast cancer patients revealed that the tumor suppressor microRNA *MIR342* is specifically lost in AA triple‐negative breast cancer (TNBC) (Loo et al. 2011). Additionally, the copy number of *MIR151* located in 8q24.3 is increased in AA PC and correlates with metastasis (Barnabas et al. 2011). In TNBC, 26 microRNAs located in fragile regions were differentially expressed between AAs and CAs, including miR‐150‐5p, miR‐200c‐3p, and miR‐205‐5p, which showed the highest fold changes in expression between the groups (Sugita et al. 2016). Furthermore, the *MIR34B* region is specifically depleted in AA cancer cells but not in CA cancer cells, leading to decreased expression and altered androgen receptor signaling in PC (Shiina et al. 2017).

### 1.5 Epigenetically Regulated microRNAs in Cancer Racial Disparities

Another layer of microRNA expression regulation involves epigenetic alterations. Three separate studies have found that the promoter regions of *MIR24*, *MIR152*, and *MIR34B* are specifically methylated in AA PC, leading to their decreased expression (Hashimoto et al. 2017; Shiina et al. 2017; Theodore et al. 2014). Another study comparing AAs and CAs in colorectal cancer (CRC) revealed that seven microRNAs (*MIR137*, *MIR2682*, *MIR9‐3, MIR663A*, *MIR6130*, *MIR548AO*, and *MIR124*) are hypermethylated in AAs, while *MIR‐34B/C* are hypermethylated in CA CRC (Wang et al. 2016). A recent study discovered that *MIR483* expression is transcriptionally suppressed in AA breast cancer patients through race‐specific histone trimethylation in the promoter region (Xing et al. 2021). Intriguingly, more than half of human microRNAs are located around CpG‐rich regions, indicating that promoter methylation plays a significant role in regulating their expression (Wang et al. 2013). This finding suggests that there is growing evidence for the epigenetic regulation of microRNAs in racial disparities.

### 1.6 Circulating and Exosomal microRNAs in Cancer Racial Disparities

MicroRNAs offer significant advantages due to their stability and small size, making them excellent candidates for biomarker research. Moreover, they have been found in small extracellular vesicles (Bayat and Sadri Nahand 2024; Bhome et al. 2018; Li et al. 2022; Sharma et al. 2021, 2019; Wu et al. 2021) and can be transferred from one cell type to another, facilitating intracellular communication. This has led to the belief that microRNAs are crucial signaling molecules in the tumor microenvironment.

A pioneering study involving 10 AA and 10 CA early‐stage breast cancer patients revealed multiple circulating microRNAs (miRs) in plasma that are specific to either AAs or CAs (Zhao et al. 2010). For example, miR‐483‐5p is specifically upregulated in AAs, while miR‐155 is downregulated in CAs. Another study using both plasma and serum samples from 220 early‐stage non‐small cell lung cancer (NSCLC) patients and 220 healthy controls found that miR‐155 is the only differential MIR detected in serum samples and is elevated in AAs (Heegaard et al. 2012). However, for plasma samples, 14 MIRs were detected to be lower (the top 2 are miR‐486 and miR‐16) in AAs in the same study. This study showed that there is an inconsistency between plasma and serum samples, which could be contributed at least in part by hemolysis of the plasma samples since miR‐486 and miR‐16 are both revealed to be highly expressed in blood cells (Pizzamiglio et al. 2017). Further studies have shown that serum miR‐101 is specifically downregulated in CA PCs (Srivastava et al. 2014), while exosomal miR‐3613 is elevated in AAs compared to CAs (Moustafa et al. 2016). A meta‐analysis combining 36 studies on 15 cancer types, involving 2920 cases and 1986 controls, revealed that circulating miR‐21 is a better diagnostic marker in Asian cancer patients compared to CAs (Wu et al. 2015). Additionally, serum exosomal microRNA miR‐1304‐3p was found to be highly expressed in AA breast cancer patients and activates cancer‐associated adipocytes to promote cancer progression (Zhao et al. 2022). Similarly, miR‐510‐5p was specifically elevated in AA breast cancer serum and activated cancer‐associated fibroblasts (King et al. 2023). A recent study (Alimena et al. 2024), analyzing serum samples from 1586 ovarian cancer patients using a custom panel of 179 highly expressed microRNAs in human serum, revealed that the expression of 66 out of 179 microRNAs in the circulation was significantly influenced by race and ethnicity.

### 1.7 Other microRNAs

In addition to the previously mentioned racial disparities in microRNAs, there are several MIRs for which the exact mechanisms leading to their differential expression remain unclear. This could be due to a complex combination of transcriptional and posttranscriptional regulations. One such MIR is *MIR29*, whose low expression is correlated with poor survival in Asian cancer patients (Qi et al. 2017). *MIR29* is one of the few MIRs that have been found to be localized and enriched in the nuclear fraction (Kriegel et al. 2012), indicating that intracellular localization may be another layer of microRNA regulation that contributes to racial disparities. Intriguingly, *MIR29* has been indicated in ductal carcinoma in situ (Deshpande et al. 2022), the precancerous noninvasive lesion form of breast cancer, suggesting that *MIR29* might be a race‐specific genetic susceptibility locus. Other MIRs, such as *MIR9*, *MIR17*, *MIR181*, *MIR182*, the MIR‐200 family, *MIR212*, *MIR221*, *MIR31*, *MIR494*, *MIR99B*, *MIR4719*, *MIR6756*, and multiple others, are found to be differentially expressed in specific races, particularly in AA (Bovell et al. 2013; Chuang et al. 2012; Das et al. 2019; Gobin et al. 2023; Guttery et al. 2018; Hashimoto et al. 2019; Huang et al. 2018; Li et al. 2014; Liu et al. 2016; Mani et al. 2023; Maxwell et al. 2015; Mitchell et al. 2017; Nassar et al. 2017; Ottman et al. 2023; Paredes et al. 2018; Pollard et al. 2018; Srivastava et al. 2013; Suresh et al. 2015; Wang et al. 2015; Xiang et al. 2018; Y. Yang, Jia, et al. 2016).

A recent study on AA patients with gynecologic malignancies, including breast invasive carcinoma, cervical squamous cell carcinoma, endocervical adenocarcinoma, ovarian serous cystadenocarcinoma, uterine corpus endometrial carcinoma, and uterine carcinosarcoma, discovered 80 differentially expressed MIRs in AAs (*N* = 305) compared to CAs (*N* = 1402) (Asare et al. 2022). The study confirmed some previously identified race‐specific MIRs, including *MIR9* and *MIR29*.

### 1.8 SNVs in microRNAs and Cancer Racial Disparities

Another important mechanism in racial disparity involves SNVs, with many SNVs found to show a racial predilection (Datta et al. 2018). However, due to the low minor allele frequency for many SNVs, a larger sample size is typically required to determine the role of a specific SNV in a particular disease. Despite this, a growing number of studies suggest that SNVs do play a role in cancer racial disparities. Advances in high‐throughput technologies, the increasing number of genome‐wide association studies, and projects like the 1000 Genomes Project now allow scientists to map genomic changes at the population level.

In the context of microRNAs, it is believed that SNVs related to microRNAs modulate the intracellular microRNA‐mRNA interaction network, contributing to cancer racial disparities. In our review, we examined (i) SNPs located in MIR targets, (ii) MIRSNPs, and (iii) isomiRs, summarizing their potential contributions to racial disparities (Table 4).

### 1.9 microRNA Target SNPs

As previously discussed, microRNAs typically bind to the 3′‐UTR of mRNAs to regulate target genes through Watson‐Crick pairing. SNPs located in the UTR regions of MIR target genes can alter the binding of specific microRNAs, contributing to cancer racial disparities. For example, studies involving 102 AA and 92 CA breast cancer patients revealed that the BRCA1 UTR SNP rs8176318 G allele is significantly higher in AAs and influences miR‐639 binding (Pelletier et al. 2011; F. Yang, Chen, et al. 2016). Similarly, the STAG1 SNP rs34149860 G allele, specific to AA CRC, affects miR‐29b binding (Datta et al. 2018). Additionally, the IL16 SNP rs1131445 TT genotype is associated with an increased risk only in AAs by influencing miR‐135 binding (Hughes et al. 2013). A meta‐analysis combining six different studies found that the KRAS SNP rs712 T allele is associated with increased cancer risk only in Chinese individuals, potentially due to altered binding of miR‐let‐7 (Ying et al. 2014). Interestingly, *MIR29* and *MIRLET7* are among the microRNAs differentially expressed based on race, as previously discussed.

### 1.10 microRNA SNPs

A more direct regulation of these SNPs on racial disparities involves SNPs within the microRNAs themselves. Among the approximately 2000 microRNAs in the human genome, 321 (~15%) carry a SNP in either the precursor, mature, or seed sequences with a minor allele frequency greater than 5%. However, only 15 of these 321 microRNAs are extensively studied, each having more than 10 related publications (Zhao et al. 2022). Intriguingly, these 15 microRNA SNPs show significant differences in minor allele frequencies across geographically and ethnically diverse populations based on data from the 1000 Genomes Project (Table 5). Notably, four out of these 15 SNPs—*MIR196A2*, *MIR202*, *MIR423*, and *MIR1908*—have been identified in a previous study as globally population‐differentiated microRNAs implicated in cancer biomarkers or diagnostics (Rawlings‐Goss et al. 2014). In the following, we summarize some of the most well‐studied MIR SNPs in cancer racial disparities.

**TABLE 5:** Top MIR SNPs and their minor allele frequencies in global populations.

MIR ID	SNP ID	Minor allele frequency				
		AFR	AMR	EAS	EUR	SAS
*MIR196A2*	rs11614913	14	38.9	54.2	41.1	25.8
*MIR499A/B*	rs3746444	16.9	13.4	14.5	19.4	26.7
*MIR149*	rs2292832	27.2	31	63.7	28.2	44.6
*MIR27A*	rs895819	48.3	37.6	28	32.2	32.4
*MIR608*	rs4919510	44	28.7	52.5	17.9	33.9
*MIR423*	rs6505162	23	54.2	81.6	44.1	55.8
*MIR1908*	rs174561	2	56.8	54.7	30.3	12.7
*MIR492*	rs2289030	0.8	16.6	28.5	5.8	10.4
*MIR605*	rs2043556	23.2	37.3	26.8	21.6	25.3
*MIR149*	rs71428439	11.1	20.2	19.9	12.7	10.7
*MIR449B*	rs10061133	4.8	6.1	26.5	10	14.2
*MIR604*	rs2368392	36.8	26.7	34.6	24.3	36.2
*MIR1307*	rs7911488	5.3	31.7	34.3	34.8	54.7
*MIR618*	rs2682818	34	11.8	25.3	14.5	28.8
*MIR202*	rs12355840	72.7	26.7	8.7	15.9	20.8

Abbreviations: AFR, African; AMR, Ad Mixed American; EAS, East Asian; EUR, European; SAS, South Asian.

For example, the SNP rs2910164, located in the *MIR146A* seed sequence, regulates both the maturation and binding affinity of *MIR146A* to its targets (Jazdzewski et al. 2008). This SNP influences cancer risk in a type‐ and race‐specific manner. The G allele is associated with a higher risk of gastric cancer in Asians but not in Caucasians (Fu et al. 2014), while the GG or GC genotype correlates with larger tumors in cervical cancer among Chinese Uygur but not Chinese Han people (Ma et al. 2015). Additionally, the CC genotype is linked to a decreased risk of PC in Asians but not in Caucasians (Mi et al. 2018). For liver cancer, no association was found in Turkish (Akkiz et al. 2011c), but the G allele showed no association in one Chinese study (Shan et al. 2013; Z. Wang, Cao, et al. 2012) but predicts risk in Asians in a meta‐analysis involving 12 studies (Peng et al. 2014). Conflicting data were seen in a meta‐analysis, which showed that the G allele predicts decreased risk for multiple cancers among Caucasians but not Asians (J. Wang, Wang, et al. 2012).

rs11614913, which is in the miR‐196A‐3p mature sequence, is so far the most widely studied microRNA SNP. *MIR196A* is implicated in various human cancers, and this SNP influences the pre‐microRNA processing and maturation. Furthermore, this locus undergoes somatic mutation in breast cancer in 14% of all patients and correlates with higher expression of *MIR196A* (Zhao et al. 2016). This SNP indeed has population‐ and cancer type‐specific effects. For example, the C allele predicts a high risk of hepatocellular carcinoma (HCC) in a Turkish population, while the T allele is associated with glioma, PC in the Asian population, and CRC in an Iranian population (Akkiz et al. 2011a; Dou et al. 2010; Haerian et al. 2018; Mi et al. 2018). In two meta‐analyses, the C allele was revealed to predict cancer risk in Asians but not in Caucasians (Kang et al. 2014; F. Wang, Ma, et al. 2012). No association for this SNP with breast cancer risk was reported in a Caucasian case–control study (Jedlinski et al. 2011), and in an HCC‐focused meta‐analysis (Peng et al. 2014) involving 12 studies solely from Asian populations, no association was observed as well.

SNP rs6505162, found in the *MIR423* precursor region, has the highest minor allele frequency among all MIR SNPs located in pre‐microRNA regions, indicating its high prevalence in human population diversity. It is implicated in multiple cancers and regulates *MIR423* maturation and processing. For example, scientists have revealed that *MIR423* rs6505162 shows a major AA genotype in AAs, an AC genotype in Caucasians, and a CC genotype in Asians (Pollard et al. 2018). The C allele is correlated with reduced maturation and processing of both miR‐423‐5p and miR‐423‐3p (Zhao et al. 2015), altering the *MIR423* targetome in a race‐dependent manner. This leads to changes in cellular behaviors, including cell proliferation, motility, metastasis, angiogenesis, autophagy, and apoptosis. Consistently, in a breast cancer study (Morales et al. 2016) from the South American population with mixed genotypes, it was shown that A‐allele carriers have a significantly increased risk in both the general population and familial breast cancers. Notably, in another study in the Jewish population, CC homozygosity at rs6505162 increased ovarian cancer risk in BRCA2‐mutated patients (Kontorovich et al. 2010). Besides, *MIR423* was reported to be an important player in obesity and regulates glucose and lipid metabolism (Ortega et al. 2013; Yang et al. 2017) and is differentially expressed in former vs. never smokers (Willinger et al. 2017). This indicates the complexity of interactions between rs6505162 and other cancer risk factors (either genetic or lifestyle or environmental exposures) in different racial/ethnic populations.

Another example is rs3746444, which is in the seed region of miR‐499A‐3p and the mature region of miR‐499B‐5p. *MIR499* is considered generally as a tumor‐suppressive microRNA, often exhibiting decreased expression in cancer cells. The G allele of the *MIR‐499* precursor displays decreased expression compared to the A allele (Ding et al. 2018). Meta‐analyses found the G allele to be associated with increased cancer risk in Asians but not Caucasians (Akkiz et al. 2011b; Chen et al. 2014; Mi et al. 2018; Shan et al. 2013; Yang et al. 2018).

Additional racially disparate MIR SNPs have been reported. For instance, the G allele for *MIR1304* is a risk allele for AA breast cancer and affects circulating *MIR1304* levels in the serum exosomes, activating cancer‐associated adipocytes (Zhao et al. 2022). The GG genotype for *MIR608* rs4919510 correlates with reduced risk of CRC death in AA but increased risk in CA patients (Ryan et al. 2012). In breast cancer, observations for *MIR106B* rs1527423 G allele, *MIR100* rs1834306 G allele, *MIR544* rs10144193 T allele, *MIR487* rs1951032 A allele, *MIR659* rs5750504 A allele with increased risk, and *MIR331* rs11107973 A allele with reduced risk were noted only in CA, while *MIR758* rs12586258 A allele and *MIR513A* rs2018562 G allele with increased risk were noted only in AA (Yao et al. 2013). Overall, we conclude that these MIR SNPs are important cancer players and exhibit effects specific to population, race/ethnicity, gender, age, and cancer type.

### 1.11 IsomiRs

Recent discoveries have unveiled further complexities in microRNAs with the identification of isomiRs—MIR variants that differ in length and sequence. This breakthrough emerged from robust analysis of high‐throughput RNA sequencing data (Landgraf et al. 2007; Morin et al. 2008). What is noteworthy is that isomiRs are unexpectedly not rare and possibly make up half of the miRNome in human cells. For each microRNA, a specific isomiR might be the dominant variant that contributes to the expression levels (Haseeb et al. 2017; Karali et al. 2016). IsomiRs are classified into four main classes: 5′ isomiRs, 3′ isomiRs, polymorphic isomiRs, and mixed‐type isomiRs, which were generated through processes like Drosha or Dicer cleavage, trimming, nucleotide addition or removal, and RNA editing, all of which can impact microRNA stability and target gene selection (Zhao 2020).

Interestingly, certain isomiRs are more abundantly expressed and serve as better biomarkers compared to their canonical forms in cancer (Wu et al. 2018). Furthermore, isomiRs exhibit race‐specific expression patterns. In breast cancer, a study (Telonis et al. 2015) identified 21 isomiRs that are differentially expressed between White and Black patients. By examining one of them, *MIR‐183*, as an example, it was revealed that each *MIR‐183* isomiR has a distinct impact on the cellular transcriptome, providing compelling evidence that supports the isomiR‐specific function in a race‐dependent manner and greatly challenges the one‐locus‐one‐MIR paradigm. In a follow‐up study, the authors further revealed that “differentially wired” mRNAs are linked with isomiRs predominantly in one of the two races and suggest that the isomiR‐mRNA associations are race and tissue‐specific (Telonis and Rigoutsos 2018). In PC, researchers detected over 3000 isomiRs, about half of which were specifically abundant in White patients (Magee et al. 2018). Another study analyzing 452 lymphoblastoid cell lines from five different population groups showed that isomiRs exhibit both race and gender‐dependent expression (Loher et al. 2014).

While the functionality and biological and clinical significance of most isomiRs remain unclear and await future research, it is evident that they will significantly impact biomarker studies in cancer research and hold great potential in uncovering new aspects of microRNA signaling in cancer initiation and progression.

### 1.12 MIR SNVs and Racial Disparities—An Under‐Explored and Evolving Field

SNVs in microRNAs are key to advancing personalized medicine. Based on the top MIR SNPs data, all of them apparently displayed ethnicity/race/population dependency and varied allele frequencies between races (Table 5), and importantly, they affect a significant percentage of patients. For example, *MIR196A2* SNP rs11614913 affects 41% of Caucasian patients, *MIR202* SNP rs12355840 affects 73% of patients with African ancestry, *MIR1908* SNP rs174561 affects 57% of Hispanic patients, while *MIR1307* SNP rs7911488 affects 35%–55% of Asian populations (Table 5). Yet, investigations about these ethnicity‐enriched SNPs have barely started.

**FIGURE 1:**
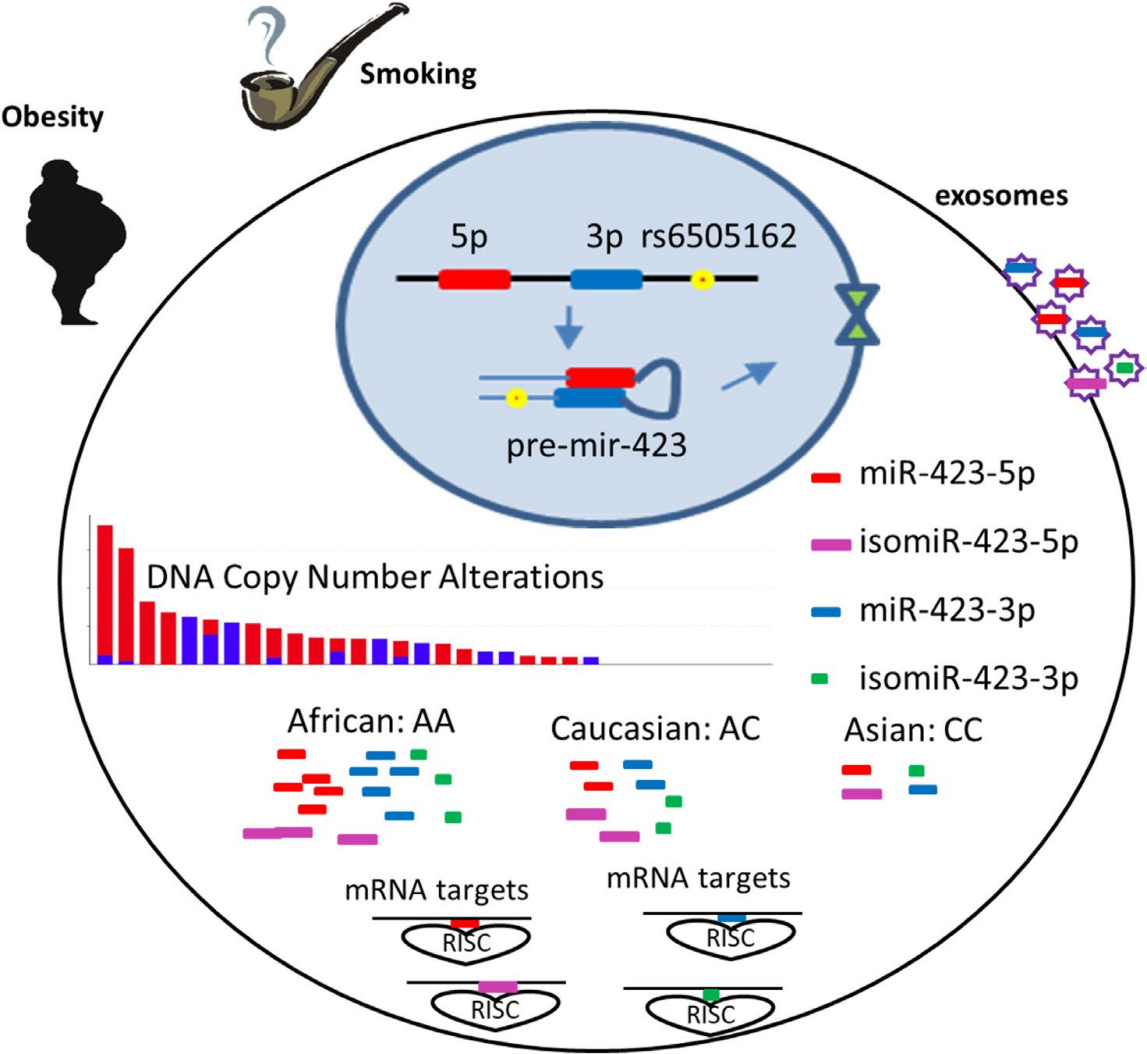
Multilayered regulation of *MIR423* in human cancer and racial disparities. It highlights copy number alterations and SNPs as well as factors including obesity and smoking affecting *MIR423* expression as well as isomiR processing and maturation leading to an altered targetome in a cancer type and race/ethnicity‐dependent manner. Exosomal *MIR423* contributes to intercellular signaling communications.

Take *MIR423* SNP rs6505162 for an example; on one hand, it is clear that it shows a major AA genotype in AAs, an AC genotype in Caucasians, and a CC genotype in Asians. On the other hand, a recent study identified significant associations between rs6505162 and *MIR423* isomiRs. The A allele was associated with both the 5′‐extension of miR‐423‐3p and the 5′‐trimming of miR‐423‐5p, and the C allele was associated with lower expression of these isomiRs (Jiang et al. 2023). Both modifications will change the MIR seed sequence and impact the *MIR423* targetome. Because this SNP is not located in the seed region but still alters MIR target selection, this study endowed non‐seed MIR SNPs with unprecedented functions. This microRNA, *MIR423*, is also regulated at the copy number level (Soh et al. 2018) as well as an exosomal microRNA itself (Xue et al. 2022; Yan et al. 2022), and influenced by both obesity and smoking, as discussed above, makes it a good example to demonstrate the multilayered regulation of microRNA in human cancer racial disparities (Figure 1).

Another example is *MIR1304* SNP rs2155248, which is also a non‐seed MIR SNP and located at the 13th base. While the T allele is the major allele, it is noted that heterozygous cells predominantly expressed isomiRs from the G allele, while TT homozygotes express a very low level of miR‐1304‐3p (Jiang et al. 2023). In addition, this microRNA is expressed at high levels in serum exosomes from AA cancer patients (Zhao et al. 2022). All these findings indicate potential effects for this SNP in microRNA processing and exosome sorting, which warrant additional investigation.

There are more than 6k SNPs on miRNA seed regions and 46.8k on microRNA precursors, out of which more than 10% (> 5.5k) are disease‐related variations (DRVs) based on GWAS studies (miRSNP v3) (Liu et al. 2021). In addition, there are thousands of isomiRs (Telonis et al. 2015). Considering the majority of these are not studied in human cancer, this field will be evolving in the foreseeable future.

## 2 Discussion

### 2.1 microRNAs and Racial Disparities Overall—Cancer and Beyond

microRNAs and their genetic variants have been implicated in racial disparities not only in cancer but also in other human diseases. Associations between microRNAs and human diseases such as diabetes (Flowers et al. 2022; Williams et al. 2019), hypertension (Arkorful et al. 2020; Dluzen et al. 2016), stroke (Akinyemi et al. 2024), cardiovascular disease (Li et al. 2018), and Hepatitis C Virus‐Mediated Liver Disease (Devhare et al. 2017) have been observed in a race/ethnicity‐specific manner. Cancer and some of these diseases exhibit a bidirectional relationship, each increasing the risk of the other. This suggests that certain race‐specific microRNAs may play crucial roles in various cellular processes and disease pathways, exerting pleiotropic effects where a single gene can manifest in multiple disease phenotypes. This aligns with the widely accepted idea that one microRNA can regulate multiple genes, allowing them to simultaneously target different genes and pathways in the context of different diseases. Interestingly, microRNAs also affect genes that are susceptible to environmental exposure or inflammation in a race‐dependent manner. For example, race‐specific microRNAs have been reported to regulate genes involved in alcohol metabolism (Rosato et al. 2019; Wakabayashi et al. 2021), tumor‐adipocyte interactions (Zhao et al. 2022), and endothelial inflammation (Sapp et al. 2021), all of which contribute to cancer initiation and progression.

### 2.2 Mechanistic Studies—Things to Consider

To fully understand the mechanisms for microRNAs in racial disparities, the following are a few things to consider. First, a major limitation in the field of microRNA research is the lack of comprehensive studies fully elucidating the regulatory control mechanisms of microRNAs. As discussed above, a significant number of microRNAs are regulated at the level of DNA copy number, epigenetic factors, SNPs, and so on; however, we should bear in mind that the complete picture might also involve transcriptional control, microRNA processing, and microRNA stability, let alone the impact of tissue/cell type‐specific expression and dynamic regulations including environmental stimuli and cellular conditions. Second, in the case of microRNA SNPs and isomiRs, to understand how each microRNA SNP/isomiR regulates disease initiation and progression, cloning of population‐enriched/allele‐specific microRNA or isomiR expression cassettes would be necessary. Third, it is now accepted that extracellular/circulating microRNAs are not only disease biomarkers but also important players in intercellular communication. These microRNAs make good candidates impacting the multilayered tumor microenvironment to explain racial disparities in initiation and progression in a cancer type‐specific manner. Fourth, microRNAs regulate gene expression through several mechanisms (Diener et al. 2024). MicroRNAs typically bind to the 3′‐UTR of target mRNAs to exert their regulatory effects, including translation inhibition and mRNA degradation; however, noncanonical binding to 5′‐UTR (Jopling et al. 2005; Orom et al. 2008) and the coding sequence (CDS) (Hausser et al. 2013) has also been reported, which does not necessarily lead to reduced gene expression at the mRNA level. Again, how geography interacts with genetics to influence health outcomes is another layer that needs to be taken into consideration to address the multidimensional puzzle in the real world. In this regard, multi‐omics analyses, including both RNA sequencing and proteome analyses, would be essential to reveal unforeseen microRNA targets and signaling axes, followed by experimental studies using both in vitro and in vivo models.

## 3 Conclusion

Precision medicine is fundamentally rooted in population genetics. While individuals exhibit unique molecular, environmental, and behavioral characteristics, it is crucial to tailor prevention and intervention strategies to these specific attributes for the diseases they have or are predisposed to. However, there remains a significant challenge: the lack of diversity and inclusion in research reference samples. Improving the representation of genetic diversity and including samples from underrepresented minorities in biobanks and datasets are essential steps toward a more comprehensive understanding of human genetics in cancer.

**FIGURE 2:**
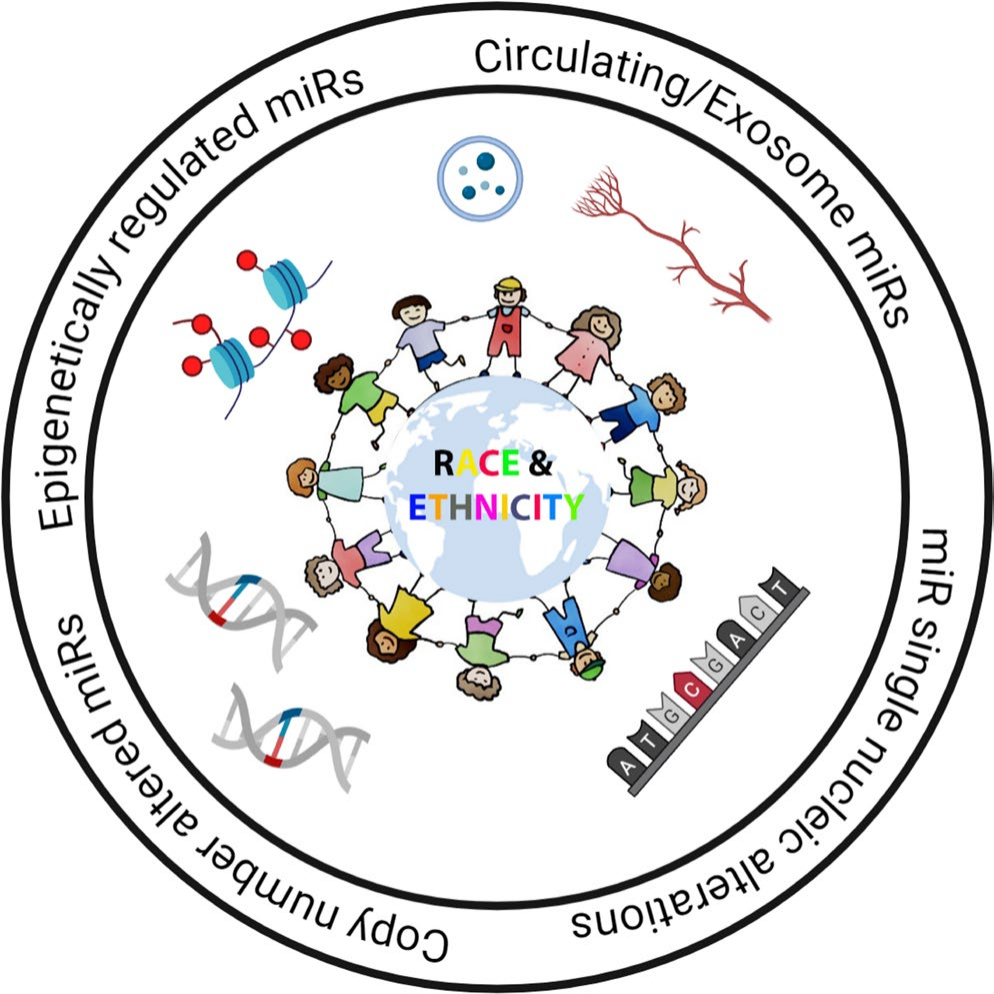
MicroRNAs and cancer racial disparities. Race/ethnicity‐specific regulation of microRNAs at multiple levels (epigenetics, copy number, single‐nucleotide polymorphisms, and circulating or exosome secretion) provides a genetic basis for cancer racial disparities.

In this focused review, we have summarized the current knowledge on microRNAs in the context of human cancer racial disparities (Figure 2). This includes microRNAs that are (i) copy number altered, (ii) epigenetically regulated, (iii) potential biomarkers in circulation or exosomes, (iv) with sequence alterations due to SNPs, and (v) of unknown mechanisms. Altogether, we review the current knowledge and progress in this field, underscoring the pivotal role of microRNAs in cancer and human health. From a population genetics or precision medicine perspective, there is a critical need to further study microRNAs as preventive biomarkers, disease determinants/drivers, or therapeutic targets in the future.

## Author Contributions

**Dan Zhao:** conceptualization (lead), data curation (lead), formal analysis (lead), investigation (lead), methodology (lead), supervision (lead), validation (lead), visualization (lead), writing – original draft (lead), writing – review and editing (lead). **Yifei Wang:** data curation (supporting), formal analysis (supporting), investigation (supporting), validation (supporting), visualization (supporting), writing – original draft (supporting), writing – review and editing (supporting).

## Conflicts of Interest

The authors declare no conflicts of interest.

## Related WIREs Articles

microRNA‐based diagnostic and therapeutic applications in cancer medicine

## Data Availability

The data used in this article are openly available. MicroRNA SNP frequency information in Table 5 is available at https://www.ensembl.org/index.html, cell line race/ethnicity information in Table 1 is available at https://www.cellosaurus.org/index.html, and patients' race/ethnicity information in Table 2 is available at https://www.cbioportal.org/.
